# Prevalence of chronic kidney disease among the high risk population in South-Western Ghana; a cross sectional study

**DOI:** 10.1186/s40697-015-0076-3

**Published:** 2015-11-03

**Authors:** Richard KD Ephraim, Sylvester Biekpe, Samuel A. Sakyi, Prince Adoba, Hope Agbodjakey, Enoch O. Antoh

**Affiliations:** Department of Medical Laboratory Technology, School of Allied Health Sciences, University of Cape Coast, Cape Coast, Ghana; Department of Molecular Medicine, School of Medical Sciences, College of Health Sciences, Kwame Nkrumah University of Science and Technology, Kumasi, Ghana; Noguchi Memorial Institute for Medical Research, Legon, Ghana

## Abstract

**Background:**

Chronic Kidney Disease (CKD) is a major global health problem. CKD is one of the most common complications of diabetes mellitus and hypertension and carries a risk of cardiovascular morbidity and mortality and progression to end-stage kidney disease.

**Objectives:**

This study sought to use the 2012 Kidney Disease Improving Global Outcomes (KDIGO) definitions to establish the prevalence and risk factors for CKD among a high risk population in the Sekondi-Takoradi metropolis.

**Design:**

Cross sectional study.

**Setting:**

Effia-Nkwanta regional and the Takoradi Government hospitals in South Western Ghana.

**Patients:**

Two hundred eight consecutive adults with diabetes, hypertension or both.

**Measurements:**

Serum creatinine and urine albumin-creatinine ratio respectively. The Chronic Kidney Disease Epidemiology Collaboration (CKD-EPI) was used to estimate glomerular filtration rate (GFR).

**Methods:**

CKD was classified according to KDIGO.

**Results:**

The prevalence of CKD was 30 %: 27 % in patients with diabetes, 22 % in patients with hypertension only and 74 % in patients with both diabetes and hypertension. GFR category G3a CKD was most prevalent stage (9 %). Albuminuria was highest among people with diabetes (39 %).

**Limitations:**

A convenience sample of patients attending clinics.

**Conclusion:**

CKD was prevalent in these high-risk patients.

## Abrégé

**Contexte:**

L’insuffisance rénale chronique (IRC) est un problème majeur de santé globale. Elle se révèle l’une des plus fréquentes complications du diabète sucré et de l’hypertension. De plus, l’IRC pose un risque accru pour les patients de souffrir, voire de mourir de cardiopathie, ou alors de voir leur état progresser vers l’insuffisance rénale terminale.

**Objectifs de l’étude:**

L’étude a cherché à établir la prévalence et les facteurs de risque de l’IRC dans la population prédisposée de la métropole de Sekondi-Takoradi (Ghana) en utilisant les définitions proposées par « Kidney Disease Improving Global Outcomes » (KDIGO) en 2012.

**Type d’étude:**

Il s’agit d’une étude transversale.

**Contexte de l’étude:**

L’étude a été effectuée sur des patients de l’hôpital régional Effia-Nkwanta et de l’hôpital gouvernemental de Takoradi, dans le sud-ouest du Ghana.

**Patients:**

L’étude était constituée d’une cohorte de 208 adultes atteints de diabète, d’hypertension ou d’une comorbidité.

**Mesures:**

Le rapport albumine-créatinine dans l’urine ainsi que le taux de créatinine sérique ont été mesurés, puis le débit de filtration glomérulaire (GFR) a été déterminé à l’aide de l’équation du « Chronic Kidney Disease Epidemiology Collaboration » (CKD-EPI).

**Méthodologie:**

L’IRC a été déterminée selon les critères de KDIGO.

**Résultats:**

À la suite de cette étude, la prévalence d’IRC a été établie à 30 % parmi les patients de la cohorte. Elle s’établissait à 27 % chez les patients atteints de diabète seulement, 22 % chez les patients atteints d’hypertension seulement et de 74 % chez les patients présentant à la fois du diabète et de l’hypertension. Le stade d’IRC (9 %) le plus prévalent était de catégorie G3a. La prévalence d’albuminurie était plus élevée chez les patients diabétiques (39 %).

**Limites de l’étude:**

Il s’agit d’un échantillon de commodité formé de patients fréquentant les deux cliniques mentionnées plus haut.

**Conclusions:**

La prévalence d’insuffisance rénale chronique était plus élevée chez ce groupe de patients considérés à haut risque.

## Background

Chronic Kidney Disease (CKD) is defined as abnormalities of kidney structure or function, present for more than 3 months, with implications for health [1, 2]. It is characterized by either decreased glomerular filtration rate (GFR) or albuminuria, or both, and carries a risk of cardiovascular morbidity and mortality and progression to end-stage renal disease (ESRD) [3]. Chronic kidney disease is thought to be prevalent in sub-Saharan Africa and to be a major public health problem [4]. Resources for recognition and management aiming at reduction in progression are limited, and resources for the treatment of ESRD severely limited [4].

Chronic kidney disease (CKD) is one of the most common complications of diabetes mellitus [5] and hypertension [5]. Screening for CKD is not routinely performed in many diabetic clinics in sub-Saharan Africa because of limited human resource, diagnostic facilities and the cost of the tests [5–7].

Several studies within sub-Saharan Africa have examined the prevalence of CKD in people at high risk, including those with diabetes and hypertension. Janmohamed et al., [7] recorded 84 % prevalence in adult outpatients with diabetes in Tanzania, and Osafo et al. [8] showed a CKD prevalence of 47 % among Ghanaian patients, mainly from the Greater Accra region, with hypertension. In addition, Sumaili et al., [9] recorded 44 % prevalence in patients with hypertension, 39 % in those with diabetes; 16 % in people with obesity and 12 % in those who had human immunodeficiency virus (HIV) or acquired immunodeficiency syndrome (AIDS). We used the 2012 guidelines of the kidney disease improving global outcomes (KDIGO) to classify CKD among patients with diabetes, hypertension and both and also identified the associated risk factors for CKD in the Sekondi-Takoradi metropolis in south western Ghana.

## Methods

### Study design and study site

A cross-sectional study was conducted at the outpatient diabetes and hypertension clinics of the Effia-Nkwanta Regional hospital (ERH) and the Takoradi Government Hospital (TGH) in the Sekondi-Takoradi metropolis between December 2012 and May 2013. These serve as the major healthcare facilities in the metropolis providing primary, secondary and tertiary healthcare services for a population of 445,000. The healthcare system is accessible to those who contribute or pay the minimum of GH 20.0 yearly premium, equivalent to about three times the daily minimum wage of GH 6.0; about 66 % of the population is covered. In Ghana, patients with diabetes or hypertension receive specialized care in teaching, regional or municipal hospitals since they are the only facilities with the capacity to diagnose and manage this condition. Sekondi-Takoradi is the administrative capital of the Western Region. It has a land area of 385 km^2^ and is located in the South-Western part of Ghana and about 242 km west of Accra, the capital city of Ghana.

### Inclusion and exclusion criteria

We enrolled eligible adult (>18 years) outpatients receiving medical care at the diabetes and hypertension clinics of the hospitals during the study period. Patients diagnosed with high blood pressure or on anti-hypertensive drugs, diabetes or both hypertension and diabetes were included in this study. We excluded patients with other kidney diseases (such as glomerulonephritis, vasculitis, kidney infection, connective tissue disease or adult polycystic kidney disease), those undergoing peritoneal or hemodialysis, and those with inflammatory bowel disease or rheumatoid arthritis. We also excluded people with known hepatitis B or C and HIV/AIDS.

### Patient screening, recruitment and data collection

We screened 382 consecutive patients with diabetes, hypertension or both who visited the outpatient department of the two hospitals for routine evaluation. Diabetes was defined as a diagnosis of diabetes or taking a hypoglycaemic drug, and hypertension as a diagnosis of hypertension or taking an anti-hypertensive drug. Information on age, gender, fasting blood glucose, body mass index (BMI), systolic blood pressure and diastolic blood pressure, medication used, duration on medication, and duration of diabetes was obtained using a pre-tested questionnaire and the patient medical records.

### Measurement of blood pressure

Trained personnel used a mercury sphygmomanometer (ACCOSON, England) with a standard or a large cuff, appropriate to the patient’s size, to measure blood pressure after patients rested for 5 min, in accordance with recommendations of the American Heart Association Council on High Blood Pressure Research [10]. We report mean values of duplicate measurements.

### Body mass index (BMI)

Height (nearest centimetre) and weight (nearest 0.1 kg), without shoes and in light clothing were measured. Participants were weighed on a bathroom scale (Zhongshan Camry Electronic Co. Ltd, Guangdong, China) and their height measured with a wall-mounted ruler. BMI was calculated by dividing weight (kg) by height squared (m^2^), and categorized according to WHO criteria into normal weight (BMI 18.5–24.9), underweight (<18.5), overweight (25.0–29.9), obese (30.0–39.9) [11].

### Blood sample collection and processing

A 4 ml venous blood sample was collected from each participant and 1 and 3 ml were dispensed into a fluoride oxalate tube and a serum gel separator tube respectively. After centrifugation at 1500 g for 3 min, the plasma and serum were stored in cryovials at −80 °C until assays were performed.

### Biochemical analysis

Plasma fasting blood sugar (FBS), serum urea and creatinine were estimated using automated chemistry analyzer (Selectra JR). Estimated glomerular filtration rate (eGFR) was calculated using the CKD-EPI equation using the coefficients for black ethnicity in all [12].

### Urine sample collection and processing

Urine protein was quantitatively estimated using the method of [13]. Estimation of urine creatinine was done using automated analyzer (ENVOY500/BT 3000 chemistry analyzer. Urine protein-creatinine ratio (uPCR) was calculated by the following formula: uPCR (mg/mmol = urine protein (mg/dl)/urine creatinine (mmol/dl). The urine protein/creatinine ratio (uPCR) was reported as mg/mmol. Resources were not available to measure albuminuria. The relationship between uACR and uPCR is not a simple one, so we did not attempt to convert between the two [14]. Instead we report uPCR in uACR categories, recognizing that this leads to overestimation of the proportions of patients with problems.

### Statistical analysis

Analysis was performed using Graphpad prism version 5.0 (GraphPad software, San Diego California USA, www.graphpad.com). Two-sample Student’s *t* test and chi-squared or Fisher’s exact test, as appropriate, and one-way analysis of variance (ANOVA) were used to compare groups. A *P*-value ≤0.05 was considered statistically significant.

### Ethical considerations

The study was approved by the University of Cape Coast institutional review board (UCC/IRB) and the committee of ethics of TGH and ERH. Written informed consent was obtained from all participants.

## Funding

Study procedures including collection of clinical data and the laboratory tests were funded by the authors.

## Results

**Fig. 1:**
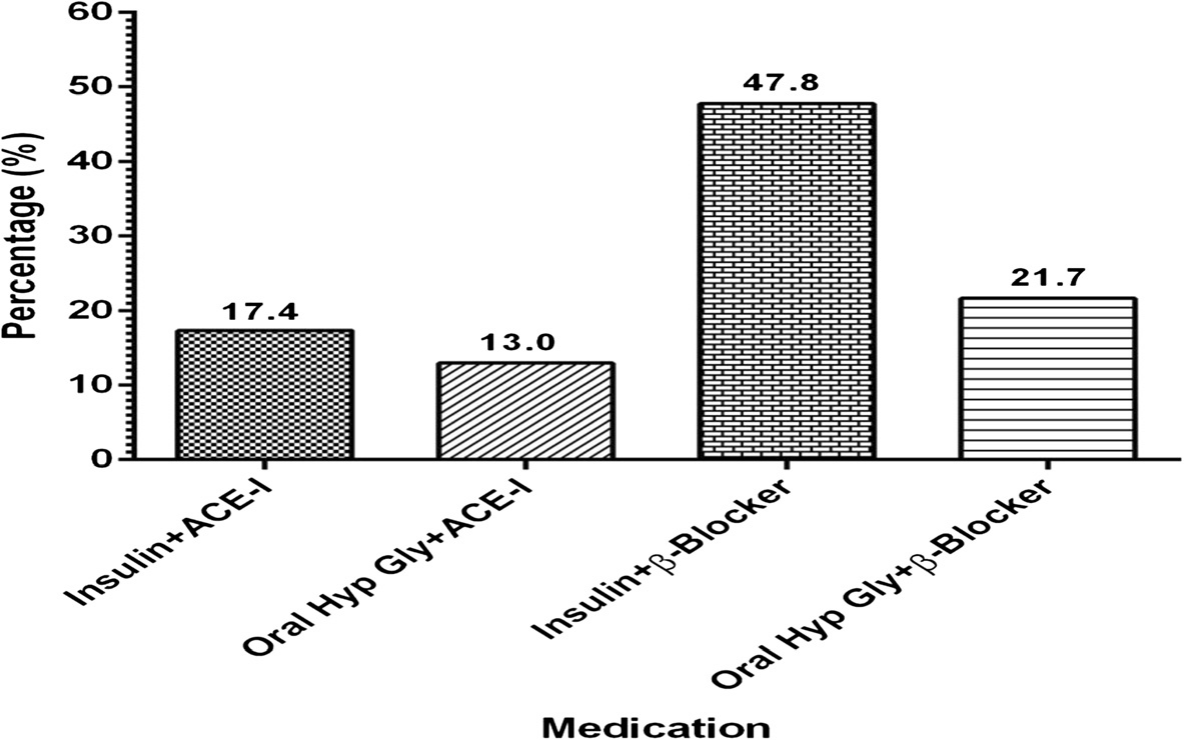
Types of medications used by participants

**Fig. 2:**
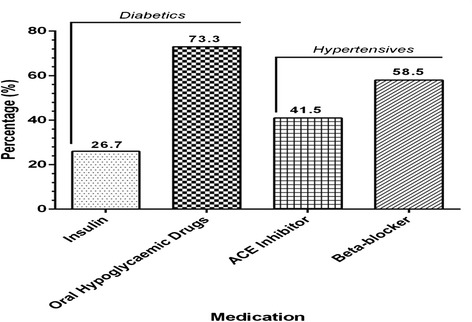
Medications used by participants with diabetes and hypertension

We screened 382 consecutive patients, of whom 76 were less than 18 years, and 67 declined to participate: of the 239 consenting participants, 208 provided both blood and urine samples (i.e., 87 % with complete data) and are the subject of this report. Mean age was 60 and 71 % were female (Table 1); blood pressure was higher in participants with known hypertension. The distribution of body weight did not vary with diagnosis (diabetes, hypertension or both) (Table 1). Figures 1 and 2 report the types of medication used by participants and the types of medications used by hypertensives and diabetics respectively.

**Table 1:** Demographic, clinical and biochemical characteristics of study participants stratified by clinical conditions

Variable	Diabetes (*n* = 120)	Hypertension (*n* = 65)	Hypertension and diabetes (*n* = 23)	*P*-value
Age (years)	60.2 ± 10.1	60.8 ± 9.5	60.7 ± 8.6	0.913
Age group (years)				
< 40	5 (4.2)	1 (1.5)	0 (0.0)	
40–49	11 (9.2)	8 (12.3)	1 (4.3)	
50–59	38 (31.7)	16 (24.6)	9 (39.1)	
60–69	44 (36.7)	27 (41.5)	9 (39.1)	
≥ 70	22 (18.3)	13 (20.0)	4 (17.4)	
Sex				
Male	32 (26.7)	21 (32.3)	7 (30.4)	
Female	88 (73.3)	44 (67.7)	16 (69.6)	0.795
Disease Duration (years)	5.4 ± 2.4	5.2 ± 2.3	6.3 ± 3.4	0.192
Disease Duration Category				
< 5	51 (42.5)	30 (46.2)	8 (34.8)	
5–10	65 (54.2)	32 (49.2)	13 (56.5)	
> 10	4 (3.3)	3 (4.6)	2 (8.7)	0.721
FBG (mmol/l)	9.7 ± 3.7	5.8 ± 1.4	13.4 ± 4.7	<0.001
SBP (mmHg)	130.3 ± 15.1	145.7 ± 22.8	160.0 ± 17.8	<0.001
DBP (mmHg)	75.1 ± 8.7	85.2 ± 10.2	94.8 ± 12.0	<0.001
BMI (kg/m^2^)	27.5 ± 5.2	27.4 ± 5.0	27.7 ± 5.6	0.966
BMI Category n(%)				
Underweight	3 (2.5)	1 (1.5)	0 (0.0)	
Normal	34 (28.3)	19 (29.2)	10 (43.5)	
Overweight	48 (40.0)	29 (44.6)	6 (26.1)	
Obese	35 (29.2)	16 (24.6)	7 (30.4)	0.672
eGFR, mL/min/1.73 m^2^	82 ± 32	76 ± 26	61 ± 39	0.012

*FBG* fasting blood glucose, *BMI* body mass index, *ACE* angiotensin converting enzyme, *eGFR* estimated glomerular filtration rate

Overall, 13 of 208 participants (6.2 %) had GFR less than 30, and 50 (24 %) had eGFR less than 60 mL/min/1.73 m^2^ (Table 2); 4 of 40 participants (10 %) had uPCR > 30 mg/mmol, and 43 (96 %) had uPCR 3–30 mg/mmol. GFR less than 30 mL/min/1.73 m^2^ and uPCR > 3 were more prevalent in those with both diabetes and hypertension than in patients with just one diagnosis (Table 3). Age and gender were similar across eGFR categories, but patients with the lowest eGFR had the highest systolic and diastolic blood pressures, and systolic blood pressure was above 140 in 17 and 26 % respectively of those in eGFR categories 3a and 3b, and in 36 % of those in eGFR category 4 (eGFR less than 30 mL/min/1.73 m^2^).

**Table 2:** Prevalence of albuminuria, estimated glomerular filtration rate (eGFR) and stages of CKD stratified by clinical conditions

Variable	Diabetes (*n* = 120)	Hypertension (*n* = 65)	Hypertension and diabetes (*n* = 23)	Total (*n* = 208)	*P*-value
uPCR					
<3 mg/mmol	102 (85.0)	52 (80)	7 (30.4)	161 (77.4)	*p* < 0.001
3–30 mg/mmol	18 (15.0)	10 (15.4)	15 (65.2)	43 (20.7)	
>30 mg/mmol	0 (0.0)	3 (4.6)	1 (4.3)	4 (1.9)	
eGFR, mL/min/1.73 m^2^ n (%)					
≥90	39 (33)	16 (25)	5 (22)	60 (29)	*p* < 0.001
60–89	54 (45)	39 (60)	5 (22)	98 (47)	
45–59	15 (13)	1 (1.5)	3 (13)	19 (9.1)	
30–44	9 (7.5)	5 (7.7)	4 (17)	18 (8.7)	
15–29	3 (2.5)	4 (6.2)	6 (26)	13 (6.2)	
<15	0 0.0)	0 (0.0)	0 (0.0)	0 (0.0)	
CKD n (%)					
Stage 1: eGFR ≥90 + Alb	1 (0.8)	1 (1.5)	1 (4.3)	3 (1.4)	0.16
Stage 2: eGFR 60–89 + Alb	4 (3.3)	3 (4.6)	3 (13.0)	10 (4.8)	
Stage 3a: eGFR 45–59	15 (12)	1 (1.5)	3 (13.0)	19 (9.1)	
Stage 3b: eGFR 30–44	9 (7.5)	5 (7.7)	4 (17)	18 (8.7)	
Stage 4: eGFR15–29	3 (2.5)	4 (6.2)	6 (26)	13 (6.3)	
Stage 5: eGFR < 15	0	0	0	0	
Total CKD, n (%)					
(Stages 1–5)	32 (27)	14 (22)	17 (74)	63 (30)	

Note to authors: the reference here should be a citation and should be to the earlier version of the guidelines that used the staging terminology

We include stages as described in earlier KDIGO guidelines (KDIGO 2013) for the purposes of comparison with other studies

*uPCR* rine protein-creatinine ratio, *eGFR* estimated GFR, *CKD* chronic kidney disease

**Table 3:** Prevalence of albuminuria and estimated glomerular filtration rate (eGFR) stratified by clinical conditions

Condition		uPCR		
		(<3 mg/mmol)	(3–30 mg/mmol)	(>30 mg/mmol)
Hypertension				
	G1	15 (23)	1 (2)	0 (0)
	G2	36 (55)	2 (3)	1 (2)
	G3a	1 (2)	0 (0)	0 (0)
	G3b	0 (0)	4 (6)	1 (2)
	G4	0 (0)	3 (5)	1 (2)
Diabetes				
	G1	38 (32)	1 (1)	0 (0)
	G2	50 (42)	4 (3)	0 (0)
	G3a	11 (9)	4 (3)	0 (0)
	G3b	3 (3)	6 (5)	0 (0)
	G4	0 (0)	3 (3)	0 (0)
Hypertension and diabetes				
	G1	4 (17)	1 (4)	0 (0)
	G2	2 (9)	3 (13)	0 (0)
	G3a	0 (0)	3 (13)	0 (0)
	G3b	1 (4)	3 (13)	0 (0)
	G4	0 (0)	5 (22)	1 (4)

Each column shows n (% within diagnosis)

No patient had G5 eGFR; G1 = eGFR ≥90 G2 = eGFR 60–89, G3a = eGFR 45–59, G3b = eGFR 30–44, G4 = eGFR 15–29

Table 3 show the distribution of patients by GFR and albuminuria categories, for those with hypertension, diabetes and both diabetes and hypertension. Overall, 30 % of participants fell into the category defined by KDIGO as ‘very high risk’: 23 % of patients with hypertension, 27 % of patients with diabetes and 74 % of those with both diabetes and hypertension.

Multivariable predictors of the presence of CKD were diagnosis category and duration on medication, both with odds ratios around 10, but not age, gender or BMI (Table 4).

**Table 4:** Multivariable associations of clinical variables with CKD in high-risk population

Variable	OR (95 % CI)	P-value
Gender		
Male^a^	Referent	
Female	1.63 (0.82–3.24)	0.167
Age group		
<40^a^	Referent	
40–49	0.88 (0.07–10.46)	0.921
50–59	2.16 (0.24–19.75)	0.496
60–69	2.41 (0.27–21.67)	0.433
≥70	2.80 (0.30–26.42)	0.369
Condition		
HPT^a^	Referent	
DM	1.33 (0.65–2.71)	0.442
DM/HPT	10.32 (3.43–31.09)	0.000
BMI n (%)		
Underweight^a^	Referent	
Normal	0.14 (0.01–1.47)	0.102
Overweight	0.14 (0.01–1.45)	0.1
Obese	0.13 (0.01–1.31)	0.083
Duration on medication		
<5^a^	Referent	
5–10	1.01 (0.54–1.87)	0.989
>10	8.96 (1.74–46.10)	0.009
Diabetes medication		
Non-diabetics	Referent	
Insulin	1.10 (0.48–2.55)	0.818
OHA	0.541 (0.28–1.05)	0.069
Anti-hypertensive medication		
Non-hypertensive	Referent	
ACE-I	0.55 (0.21–1.45)	0.225
β-blocker	0.51 (0.22–1.20)	0.124

^a^refers to the referent

## Discussion

We identified a prevalence of CKD in patients with hypertension of 22 % and in patients with diabetes of 27 %. In patients with both hypertension and diabetes, the prevalence was 74 %, and 26 % had category G4 CKD. Clinical factors associated with a greater risk of CKD were the presence of both hypertension and diabetes, and duration on medication (antidiabetic and antihypertensive).

Osafo and colleagues [8] reported a 47 % prevalence of CKD among patients with hypertension in Ghana, in a multicenter study conducted predominantly among people with hypertension in the Greater Accra area (known for a high prevalence of hypertension). This is higher than the overall prevalence of 30 % among our study participants, and 22 % in people with hypertension. The difference may, in part, be owing to our having used the CKD-Epi [12] equation rather the MDRD equation, which was used in the study by Osafo and colleagues. MDRD is known to overestimate the prevalence of CKD compared with CKD-Epi, and this has also been shown by Kitiyakara and colleagues in their study of the high risk population in South East Asia [15]. In all patients with diabetes (with or without hypertension) we observed a prevalence of 48 %, which is lower than the 80 % prevalence observed among African adults with diabetes in a cross-sectional study conducted in Tanzania by Janmohamed and colleagues [7]. Again this is not directly comparable, with differences arising from their use of Cockroft-Gault equation to calculate the eGFR (which overestimates true GFR and underestimates prevalence); and urine albumin concentration as a measure of proteinuria (which is the recommended method of assessing proteinuria; our use of uPCR overestimates prevalence) but both these differences would result in a tendency for our prevalence by our methods to be higher than by their methods; so it may be that true differences exist. However, neither study used IDMS calibrated creatinine measurement and the direction of biases resulting from this limitation cannot be assessed.

Osafo and colleagues [8] observed a 51 % prevalence of CKD in patients with coexistent diabetes and hypertension, based on data from 712 participants in a multicenter study in Accra, Ghana, and we observed 74 % prevalence of CKD in our participants. Since their use of the MDRD formula would have biased their findings towards a greater incidence of CKD, it is possible that the prevalence is truly higher in this group in Ghana.

Good blood pressure control and ACE inhibitors are known to have a reno-protective effect, particularly in people with albuminuria [16]. In our study, 30 % overall, 11.1 % of people with diabetes and hypertension, 9.2 % of people with albuminuria and hypertension received ACE inhibitor therapy. We are unable to determine from our study to what extent this relatively low prevalence represents true contraindications or previous adverse effects, or whether this is a possible missed treatment opportunity that results from the economic costs of the drug (most of which are borne by the patients) or a reluctance on the part of physicians to prescribe ACE inhibitors without access to repeated monitoring of renal function (laboratory tests are paid for by the patient).

Our study has several limitations. First our findings cannot be generalized to other low income and low resource countries because it was not community based and was conducted within a population at risk of developing CKD with genetic and cultural differences. Further, there may be differences in the practices that lead to a patient being identified as having hypertension or diabetes, and differences in access to treatments for, and monitoring of those conditions. The study was conducted in the Sekondi-Takoradi metropolis. It is likely that there would be significant variation in prevalence rates in other urban and rural towns in the Western region and across Ghana as a whole. This study is also limited by the small sample size, use of the single measurement of serum creatinine (whereas to truly fulfill definitions of CKD, two measurements at least 3 months apart are needed), and by our lack of standardization of serum creatinine to isotope mass dilution spectrophotometry (IMDS). Third, although the CKD-EPI eGFR equation has been used in previous studies in this population [8, 18, 19] it has not been validated for use in the black Ghanaian population. Strengths of our study are the consecutive sampling and completeness of data collection.

## Conclusion

CKD was detected among 30 % of this high-risk population. Further research is needed into optimal approaches to screening and treatment, including research on the effects of lowering economic barriers to known effective treatments. This is particularly important in resource-constrained practice settings such as ours, because the impact of the development of end-stage renal disease when dialysis cannot be provided is so much greater.
